# Expression of stem cell factor in gastrointestinal stromal tumors: Implications for proliferation and imatinib resistance

**DOI:** 10.3892/ol.2012.1019

**Published:** 2012-11-09

**Authors:** XIAO-WEI HOU, CHEN-GUANG BAI, XIAO-HONG LIU, CEN QIU, LING HUANG, JING-JING XU, DA-LIE MA

**Affiliations:** 1 Department of Pathology, Changhai Hospital, Second Military Medical University, Shanghai 200433;; 2 Department of Oncology, 401 Hospital of PLA, Qingdao, Shandong 266071;; 3 Institute of Cardiothoracic Surgery, Changhai Hospital, Second Military Medical University, Shanghai 200433, P.R. China

**Keywords:** stem cell factor, gastrointestinal stromal tumor, KIT, imatinib resistance

## Abstract

KIT autophosphorylation caused by mutation of *KIT* is considered to be a critical mechanism for the oncogenesis of gastrointestinal stromal tumors (GISTs). However, little is known regarding whether stem cell factor (SCF), the KIT ligand, is able to induce the proliferation of GIST cells by activating the wild-type KIT receptor in GISTs. Imatinib, a tyrosine kinase inhibitor, has been demonstrated to be effective as treatment for the majority of GISTs. However, primary resistance to imatinib in GISTs with wild-type *KIT* and acquired resistance in GISTs with mutant *KIT* are becoming increasingly significant problems. The aims of this study were to detect the expression and function of SCF in 68 GIST samples, and to explore the relationship between SCF activity and imatinib resistance using immunohistochemical staining and western blot analysis. Results showed abundant expression of SCF in GISTs and demonstrated that SCF is capable of enhancing GIST cell proliferation. Similar to its ineffectiveness in wild-type GISTs, imatinib also failed to inhibit SCF-induced KIT activation in GISTs with mutant KIT. We also found increased SCF expression in GIST cells treated with imatinib. Overall, our results indicated that SCF-induced KIT activation is a novel essential pathway for the proliferation of GISTs. Imatinib was not able to inhibit the activity of SCF, while it promoted the expression of SCF, which may have contributed to acquired imatinib resistance.

## Introduction

KIT is a transmembrane glycoprotein that belongs to the type III tyrosine kinase receptor family (1). Upon binding its ligand, stem cell factor (SCF), the KIT receptor dimerizes and initiates a signal transduction phosphorylation cascade that results in the regulation of cell growth. SCF is encoded by the *Steel* gene and is present in both membrane-bound (mSCF) and soluble (sSCF) forms (2). KIT that has been activated by mSCF is considered to be stable and maintains its activity (3). The SCF/KIT system plays a key role in the differentiation and proliferation of the interstitial cells of Cajal (ICCs) and hematopoietic cells (4). The SCF/KIT system is also involved in cell proliferation in certain tumors, including mast cell leukemia, seminoma and malignant melanoma, as well as lung, small cell, breast, gastric, colon, cervical and ovarian cancer (5–10).

Gastrointestinal stromal tumors (GISTs) are the most common mesenchymal neoplasm of the gastrointestinal tract and they originate from the ICCs or their precursor cells (11). GISTs are defined as tumors that are typically immunoreactive for KIT. Unlike the SCF/KIT tumors mentioned previously, ∼75–80% of GISTs have a gain-of-function mutation in the *KIT* proto-oncogene encoding the KIT protein (12–15). These mutations lead to constitutive oncogenic signaling in the absence of SCF. Uncontrolled KIT activity results in the oncogenesis and proliferation of GISTs. However, it has been demonstrated that 94% of GIST mutations are heterozygous, i.e., wild-type KIT remains present in the majority of GISTs (16). It is not yet known whether these wild-type KIT are capable of being activated by their ligand, SCF, and are involved in the proliferation of GISTs.

Imatinib, a small molecule tyrosine kinase inhibitor, has been demonstrated to be effective in the treatment of recurrent or metastatic GISTs by inhibition of KIT activation. Findings of a previous study confirmed that KIT activation is a ubiquitous feature of GISTs, even in the absence of *KIT* mutations (14). However, based on certain clinical trials, the best response rates to imatinib have been observed in GISTs with *KIT* mutations (12,17–18). GISTs with wild-type *KIT* have demonstrated primary resistance to imatinib, and a considerable proportion of GISTs with mutant *KIT* have demonstrated acquired resistance at a later stage (19). These clinical trials revealed that imatinib was not capable of effectively inhibiting wild-type KIT activation in GISTs lacking a *KIT* mutation. If SCF is able to function as the ligand that activates wild-type KIT in heterozygous GISTs, we hypothesize that imatinib is also likely to fail to inhibit wild-type KIT activation.

In the present study, we examined the expression of SCF in GISTs, analyzed the relationship between SCF expression and the proliferative activity of GIST cells, and studied the role of SCF in the proliferation of GIST cells. We verified our theory by examining KIT activation in SCF-stimulated GIST cells pretreated with imatinib. We also observed the level of SCF expression in GIST cells following treatment with imatinib.

## Materials and methods

### Patient samples

Clinical samples were obtained with informed consent. A total of 68 GIST samples were included in the study. All cases were confirmed as GIST by at least two pathologists. The tumor size, number of mitotic cells in 50 high-power fields (HPF) and Ki-67 index were observed by hematoxylin and eosin (HE) staining or immunohistochemistry. For the histological and immunohistochemical analyses, tissue specimens were fixed in 10% formalin and embedded in paraffin. Core tissue biopsies (2 mm in diameter) were taken from individual paraffin-embedded tumor tissues and arranged in new recipient paraffin blocks using a Tissue Microarrayer (Beecher Instruments, Silver Spring, MD, USA). For each tumor, the representative tumor areas were selected using HE staining. For western blot analysis and mutation detection, 21 fresh tissue specimens were frozen and stored at −80°C.

### Immunohistochemical detection

Immunohistochemical staining was performed using anti-SCF antibody (rabbit monoclonal; 1:50 dilution; Abcam, Cambridge, MA, USA), anti-KIT antibody (rabbit polyclonal; 1:500 dilution; DakoCytomation, Glostrup, Denmark) and anti-Ki-67 antibody (mouse monoclonal; MIB-1; 1:200 dilution; DakoCytomation). The TMA sections (4 *μ*m) were deparaffinized and boiled in 10% citric acid buffer solution (pH 6.0) for 20 min for antigen retrieval. Following blocking of endogenous peroxidase activity with 3% hydrogen peroxide, specimens were incubated with primary antibodies at 4°C overnight. Bound antibodies were detected by a peroxidase-labeled, polymer-conjugated secondary antibody (EnVision HRP; DakoCytomation), and subjected to peroxidase staining using diaminobenzidine (DAB) as a substrate. Slides were counterstained with hematoxylin. The Ki-67 labeling index was calculated as the percentage of Ki-67-positive cells among all tumor cells in 5 HPF.

### Detection of KIT mutations

Genomic DNA of fresh or paraffin-embedded tissues was extracted using a standard proteinase-K digestion/phenol-chloroform procedure. *KIT* exons 11 and 9 were amplified using the following primer sequences and annealing temperatures: Exon 11, forward: 5′-CCAGAGTGCTCTAATGACTG-3′ and reverse: 5′-AGC CCCTGTTTCATACTGAC-3′ at 60°C; exon 9, forward: 5′-GCCACATCCCAAGTGTTTTATG-3′ and reverse: 5′-GAG CCTAAACATCCCCTTAAATTG-3′ at 56°C. Sequencing analysis was performed directly on the PCR products.

### Cell isolation and primary cell culture

**Figure 1.**
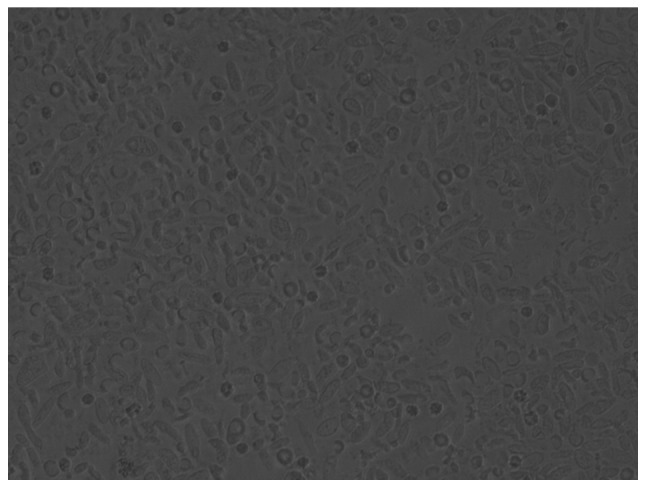
Primary cultures of gastrointestinal stromal tumor (GIST) cells.

Fresh GIST tissues were minced with scissors, washed twice with phosphate-buffered saline (PBS), and then ground into single-cell suspensions by filtering through the sieve with 200 *μ*m mesh. After washing in cold PBS, cell pellets were resuspended in RPMI-1640 medium supplemented with 10% fetal calf serum (FCS; Gibco, France) and seeded onto culture dishes (Fig. 1). Cells were cultured overnight prior to analysis. Simultaneously, all the cells that had been isolated from GIST tissue were analyzed for *KIT* mutations (Table I).

### In vitro cell proliferation assay and drug testing

GIST cells were FCS-starved for 4 h and then cultured in the appropriate culture medium with 100 ng/ml recombinant human SCF (rhSCF, Peprotech, Inc., Rocky Hill, NJ, USA) for 72 h. Viable cells were measured with the Cell Counting kit 8 (Wako, Osaka, Japan) according to the manufacturer’s instructions. GIST cells were FCS-starved for 4 h, stimulated by rhSCF for 0, 5, 15, 30, 60 or 120 min, and then harvested for KIT phosphorylation detection by western blot analysis. SCF expression levels were detected in GIST cells treated with or without imatinib (Novartis Pharma, Basel, Switzerland) for 72 h by western blot analysis. KIT phosphorylation levels were observed in GIST cells treated with imatinib for 90 min, prior to stimulation with 100 ng/ml rhSCF for 10 min.

### Western blot analysis

Frozen GIST samples were calibrated and homogenized in lysis buffer (20 mM Tris, 150 mM NaCl, 1 mM othovanadate, 10 mM NaF, 1 mM PMSF, 0.5 *μ*g/ml leupeptin, 1 *μ*g/ml pepstatin, 10 KIU/ml aprotinin and 1% triton X-100). Lysates were rocked at 4°C for 30 min and then centrifuged at 12,000 rpm for 15 min. Supernatant protein concentrations were measured using a BAC Protein Assay kit (Merck KGaA; Darmstadt, Germany), and 50 *μ*g of protein were separated by 8 or 12% SDS-polyacrylamide gel electrophoresis and transferred to a polyvinylidene difluoride membrane. The membrane was blocked for 60 min at room temperature with 5% skimmed milk or bovine serum albumin (BSA) and then reacted with anti-KIT antibody (DakoCytomation), anti-SCF antibody (Abcam), or anti-c-kit (phospho Y703) (Abcam), as the primary antibody. Peroxidase-labeled anti-rabbit IgG was used as the secondary antibody. The Western Lightning chemiluminescence reagent (Santa Cruz Biotechnology, Inc.; Santa Cruz, CA, USA) was used for the detection of proteins.

## Results

### SCF expression in GISTs and its correlation with tumor proliferation

**Figure 2.**
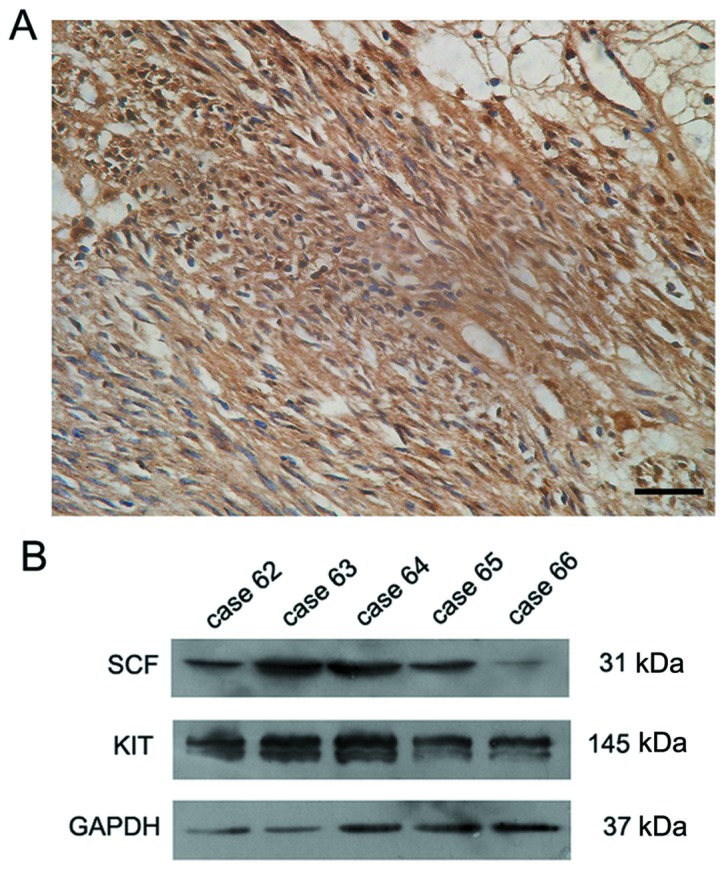
Expression of SCF and KIT in gastrointestinal stromal tumors (GISTs) by immunohistochemistry and western blot analysis. (A) Expression of stem cell factor (SCF) protein in GISTs revealed by immunohistochemistry. SCF staining areas are on the membrane and in the cytoplasm of cells. (B) Western blot analysis of SCF and KIT protein. The bands at 31 and 145 kDa correspond to SCF and KIT protein, respectively.

The expression of KIT and its ligand SCF were detected by immunohistochemical staining in 68 GIST samples. All GIST samples demonstrated KIT positivity. Expression of SCF was observed in 52 cases (Fig. 2A). The SCF-positive cases presented moderate or strong staining in the membrane and cytoplasm of GIST cells. Expression of SCF was further demonstrated by western blot analysis in fresh GIST tissues that stained positive for SCF by immunohistochemistry. As shown in Fig. 2B, a positive signal was detected at 31 kDa that corresponded to the membrane-bound form of SCF in 17 out of 21 cases. In those 17 tumors, the signal for KIT, which has a molecular weight of 145 kDa, was also confirmed in the tumor tissues (Fig. 2B).

We then examined the association between SCF expression and the proliferative activity of tumors. The Ki-67 index and the number of mitotic cells in 50 HPF were used to evaluate the proliferation potential of the GIST cells. The expression rate of Ki-67 and the number of mitotic cells in SCF-positive cases were significantly higher than those of SCF-negative cases (P<0.001 and P<0.05, respectively; Mann-Whitney U test; Table II).

### Correlation between c-kit mutations and SCF expression

A total of 54 GIST samples were investigated for mutations in the *KIT* proto-oncogene (exons 9 and 11). Exons 9 and 11 have been demonstrated to be frequently mutated in GISTs. DNA sequencing results obtained for all 54 samples are listed in Table III. The correlation of *KIT* mutations with SCF expression was analyzed by a McNemar’s test. No correlation was observed between the presence of *KIT* mutations and the expression of SCF (P>0.05).

### Proliferation of GIST cells stimulated by SCF

**Figure 3.**
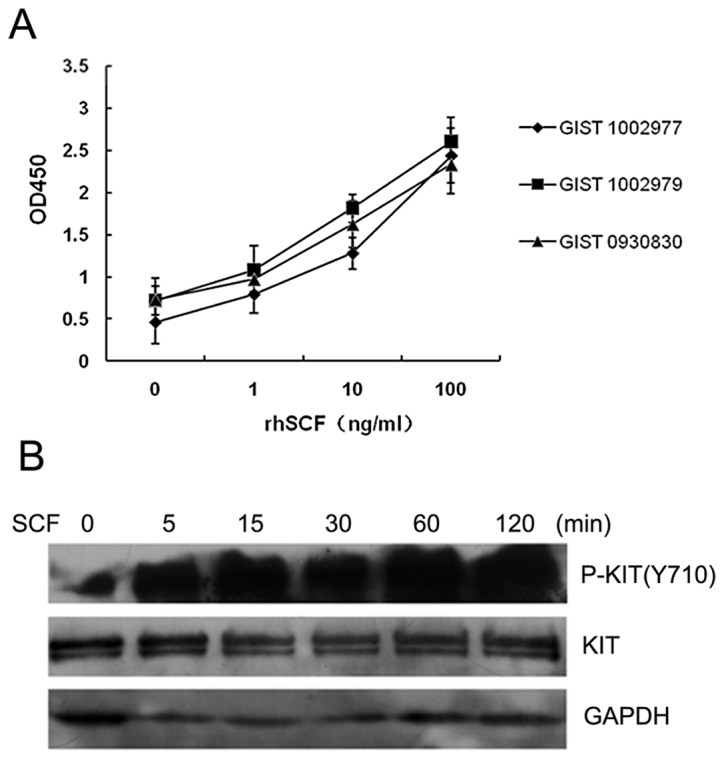
Gastrointestinal stromal tumor (GIST) cell responses to recombinant human stem cell factor (rhSCF). (A) Three representative samples of primary GIST cells proliferated in response to SCF in a dose-dependent manner. (B) The level of KIT phosphorylation in SCF-stimulated cells was higher than that of unstimulated cells.

To investigate the function of SCF/KIT signaling in GIST cells, primary GIST cells were incubated for 72 h with SCF at concentrations of 0, 1, 10 and 100 ng/ml, and their viability and rate of multiplication were determined by a cell count assay. The primary GIST cells from all three patients examined proliferated in response to SCF in a dose-dependent manner (Fig. 3A). We subsequently examined KIT activation in GIST cells following cultivation with or without SCF. The levels of KIT phosphorylation in SCF-stimulated cells were higher than those of unstimulated cells (Fig. 3B). These results suggest that SCF, as the ligand of KIT, is capable of activating its receptor in GIST cells. In addition, the results suggest that the SCF/KIT signaling pathway plays a key role in the proliferation of GIST cells.

### Effect of imatinib treatment on SCF expression and SCF-stimulated KIT activation in GIST cells

**Figure 4.**
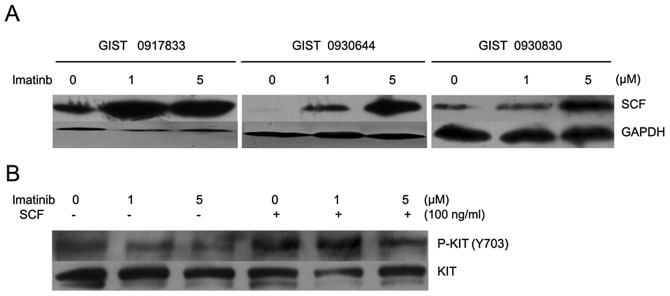
Effect of imatinib treatment on stem cell factor (SCF) expression and SCF-stimulated KIT activation in GIST cells. GIST cells were treated with or without imatinib and SCF. (A) SCF expression increased in three GIST samples treated with imatinib. (B) Imatinib failed to suppress the SCF-mediated activation of KIT.

Treatment with imatinib has been shown to result in an increased SCF concentration in the serum of GIST patients (20). To test the effect of imatinib treatment on SCF expression of primary GIST cells *in vitro*, FCS-starved GIST cells were treated with imatinib for 72 h and then analyzed by western blot analysis for SCF expression. The SCF signal was stronger in imatinib-treated compared with untreated GIST cells (Fig. 4A). These results suggest that imatinib is capable of increasing endogenously produced SCF in cultured GIST cells. We then examined the inhibitory effects of imatinib on KIT activation that has been induced by stimulation with exogenous SCF. GIST cells were pre-treated with or without imatinib and then stimulated with 100 ng/ml SCF. Following the 100 ng/ml SCF stimulation, higher levels of KIT phosphorylation remained present in GIST cells. An increased dose of 5 *μ*M imatinib was not able to inhibit KIT phosphorylation in SCF-stimulated cells *in vitro* (Fig. 4B). These results suggest that imatinib may not effectively inhibit KIT phosphorylation that has been induced by its ligand (SCF).

## Discussion

In this study, we observed abundant SCF expression in GIST tissues, which suggested an autocrine mechanism of SCF. Western blot analysis verified that the SCF protein expressed in GISTs is predominantly present as the membrane-bound form, which has the ability to stably and continuously activate its receptor, KIT. Our data also demonstrated co-expression of SCF and KIT in GISTs, and SCF expression was significantly correlated with an increased proliferative potential of GISTs. A previous study (21) also SCF expression in GISTs, as well as a larger tumor size and higher MIB-1 index in SCF-positive cases. The present data, together with these findings, suggest that SCF may be a potential marker for GIST proliferation.

There are two mechanisms of activation of KIT in malignant tumors. One is autophosphorylation of KIT due to gain-of-function mutations of the c-kit gene, and the other is ligand-dependent activation. The present study did not detect a correlation between SCF expression and the status of *KIT* mutation in GISTs. These data suggest that SCF-induced KIT activation is an independent mechanism in GISTs, regardless of KIT autophosphorylation. A previous study (22) demonstrated that the fraction of activated KIT was not correlated with the fraction of mutant KIT in GISTs. The absence of a correlation may be explained by ligand-dependent SCF/KIT signaling in GISTs.

SCF stimulation of GIST544 cells, which express a heterozygous c-kit exon 9 mutation, induces stronger KIT phosphorylation (23). SCF treatment of GIST882 cells, which contain a homozygous c-kit exon 13 mutation, does not induce high levels of KIT phosphorylation (24). One isoform of KIT that contains a 4 amino acid sequence, GNNK, has been demonstrated to be the most abundant isoform in GISTs (25). Following stimulation with SCF, wild-type GNNK-negative KIT induced an improvement in cell survival and stronger proliferation (26). In the present study, we also demonstrated that SCF was capable of inducing GIST cell proliferation *in vitro*. Additionally, a high level of KIT phosphorylation in SCF-stimulated cells was observed. All GIST cells were verified to contain heterozygous, functional mutations of c-kit. Thus, this study has demonstrated that SCF may activate wild-type KIT in GISTs and participate in the activation of heterozygous KIT mutants.

Notably, the results of the present study demonstrated increased SCF expression levels in imatinib-treated GIST cells. Tumor growth is complicated and multiple signaling pathways are involved in the survival and proliferation of tumor cells. Therefore, it is possible that in GISTs, after activation of the mutant KIT is inhibited by imatinib, other compensatory signals may become involved in maintaining tumor cell survival. As a result, increased SCF expression levels may be a response that facilitates preservation of the wild-type KIT signaling in GISTs. A previous study (27) demonstrated that mutant KIT was mainly retained within the endoplasmic reticulum and Golgi compartments in an immature constitutively phosphorylated form, whereas wild-type KIT was expressed at the plasma membrane in a mature non-phosphorylated form. Imatinib-induced inhibition of the phosphorylation of mutant KIT proteins resulted in the restoration of KIT expression at the cell surface. Our data, together with those findings, suggest that increased expression of SCF and KIT at the cell surface due to imatinib treatment results in abundant SCF/KIT signaling activation. Although the inhibitor imatinib blocked the ligand-independent signaling of KIT, it simultaneously enhanced the ligand-dependent signaling. Negri *et al*(28) also demonstrated that the surgical samples of imatinib-treated GISTs were characterized by high expression levels and activation of the wild-type KIT receptor, together with high expression levels of its ligand. These findings provide a favorable compliment to our study *in vivo*.

In this study, we verified the inefficiency of imatinib for the inhibition of KIT phosphorylation stimulated by SCF *in vitro*. In clinical studies, it has been confirmed that GISTs with no KIT mutations demonstrate resistance to imatinib. A clinical study (29) also found that there was no clinical efficacy of imatinib in uveal melanomas expressing SCF/KIT without mutations. These results suggest that the ligand-dependent activation of KIT is likely to have primary resistance to imatinib, while the stronger ligand-independent activation of wild-type KIT due to imatinib treatment may contribute to the development of acquired resistance.

In conclusion, the expression of SCF suggests an autocrine mechanism in GISTs. It is likely that ligand-independent KIT activation due to gain-of-function mutations in the *KIT* gene is the main mechanism of GIST oncogenesis, whereas the ligand-dependent activating mechanism is the crucial reason for tumor proliferation. The activation of SCF/KIT signaling may be a considerable contributing factor in imatinib resistance. Our study suggests that the simultaneous inhibition of ligand-dependent and ligand-independent activation of KIT may be a more effective strategy for GIST therapy.

## Figures and Tables

**Table I.** Details of GIST primary cultures.

GIST case no.	Age (years)	Origin	Tumor size (cm)	*KIT* mutation
0917833	53	Stomach	6.0	Exon 11 DEL557–558
0919049	41	Stomach	3.0	Exon 11 DEL555–558
0919298	68	Stomach	2.6	Exon 11 DEL557–558
0930644	37	Small intestine	2.0	Exon 9 INS502–503
0930830	58	Small intestine	8.0	Exon 9 INS502–503
1002977	56	Small intestine	11.0	Exon 9 INS502–503
1002979	59	Stomach	7.5	Exon 11 DEL576

All patients were male. GIST, gastrointestinal stromal tumor; DEL, deletion; INS, insertion.

**Table II.** Number of mitotic cells and Ki-67 index in SCF-positive cases compared with SCF-negative cases.

	No. of SCF-positive cases	No. of SCF-negative cases	P-value
No. of mitotic cells (per 50 HPF)			0.049
≤10	36	15	
>10	16	1	
Ki-67 index (%)			0.001
<5	15	12	
5–10	23	4	
>10	14	0	

**Table III.** KIT mutation details of the 54 cases of GIST.

Case no.	Gender	Age (years)	SCF	Origin	Tumor size (cm)	*KIT* mutation
1	F	40	−	Small intestine	4.0	Wt
2	M	36	+	Stomach	5.0	Wt
3	M	61	+	Stomach	5.5	Exon 11 V560D
4	M	43	+	Stomach	5.0	Exon 11 DEL555–556
8	F	50	+	Small intestine	5.0	Wt
9	F	56	+	Stomach	4.5	Exon 11 DEL556–582
10	F	72	+	Stomach	2.0	Wt
13	M	59	−	Stomach	4.0	Wt
15	M	34	+	Small intestine	3.5	Exon 9 INS502–503
16	F	49	+	Stomach	4.0	Exon 11 DEL555–558
17	F	52	+	Unknown	9.0	Exon 9 INS502–503
18	M	43	+	Small intestine	3.5	Exon 11 DEL553–554
19	F	78	−	Stomach	5.0	Exon 11 DEL555–558
20	F	61	+	Small intestine	5.0	Exon 11 DEL555–559
21	F	59	+	Stomach	3.0	Wt
22	M	49	+	Small intestine	2.5	Wt
23	F	53	+	Stomach	2.5	Wt
27	M	63	−	Stomach	5.0	Exon 11 V559D
28	M	48	+	Stomach	3.0	Exon 11 DEL565–572
29	F	39	+	Stomach	18.0	Exon 11 INS577–579
30	F	48	+	Small intestine	12.0	Wt
31	M	71	+	Stomach	5.5	Wt
32	M	53	+	Rectum	6.0	Exon 11 W557R
33	M	45	+	Small intestine	2.0	Wt
34	F	73	+	Stomach	6.0	Exon 11 V559D
37	F	57	+	Stomach	6.5	Wt
38	M	54	−	Small intestine	6.0	Exon 11 DEL579
40	M	68	−	Small intestine	7.0	Exon 11 DEL557–558
41	F	48	+	Stomach	3.0	Exon 11 DEL557–558
42	F	73	−	Stomach	2.5	Exon 11 DEL555–557
43	M	50	+	Stomach	4.0	Wt
45	F	37	+	Small intestine	3.0	Exon 11 INS557–582
47	F	51	+	Small intestine	2.5	Exon 9 INS502–503
48	F	49	+	Stomach	5.0	Exon 11 INS577–582
49	M	78	+	Stomach	14.0	Exon 11 DEL555–558
50	F	41	+	Unknown	8.0	Exon 11 V560D
53	M	77	+	Stomach	5.5	Exon 11 V559D
54	F	55	+	Stomach	22.0	Exon 11 DEL555–559
55	F	82	+	Small intestine	3.5	Exon 11 DEL553–554
56	F	55	+	Small intestine	5.0	Exon 9 INS502–503
57	F	84	−	Stomach	5.0	Wt
58	F	55	+	Stomach	4.0	Exon 11 DEL555–558
59	M	60	+	Stomach	2.8	Exon 11 DEL565–572
60	F	75	+	Stomach	6.0	Exon 11 INS577–579
61	F	63	+	Small intestine	3.0	Exon 9 INS502–503
62	M	47	+	Stomach	3.0	Exon 11 DEL576
63	M	35	+	Stomach	6.5	Exon 11 INS575–582
64	M	60	+	Small intestine	3.2	Exon 9 S451C
65	F	59	+	Stomach	14.0	Exon 11 DEL557–558
66	M	75	−	Stomach	15.0	Exon 11 DEL550–558
67	M	53	+	Stomach	6.0	Exon 11 DEL579
68	M	29	−	Rectum	5.0	Wt

GIST, gastrointestinal stromal tumor; SCF, stem cell factor; DEL, deletion; INS, insertion; Wt, wild-type.

## References

[1] Rousset D, Agnès F, Lachaume P, André C, Galibert F (1995). Molecular evolution of the genes encoding receptor tyrosine kinase with immunoglobulin like domains. J Mol Evol.

[2] Anderson DM, Lyman SD, Baird A, Wignall JM, Eisenman J, Rauch C, March CJ, Boswell HS, Gimpel SD, Cosman D (1990). Molecular cloning of mast cell growth factor, a hematopoietin that is active in both membrane bound and soluble forms. Cell.

[3] Miyazawa K, Williams DA, Gotoh A, Nishimaki J, Broxmeyer HE, Toyama K (1995). Membrane-bound steel factor induces more persistent tyrosine kinase activation and longer life span of c-kit gene-encoded protein than its soluble form. Blood.

[4] Cohen PS, Chan JP, Lipkunskaya M, Biedler JL, Seeger RC (1994). Expression of stem cell factor and c-kit in human neuroblastoma. The children’s cancer group. Blood.

[5] Hassan S, Kinoshita Y, Kawanami C, Kishi K, Matsushima Y, Ohashi A, Funasaka Y, Okada A, Maekawa T, He-Yao W, Chiba T (1998). Expression of proto-oncogene c-kit and its ligand stem cell factor (SCF) in gastric carcinoma cell lines. Dig Dis Sci.

[6] Hines SJ, Organ C, Kornstein MJ, Krystal GW (1995). Coexpression of the c-kit and stem cell factor genes in breast carcinomas. Cell Growth Differ.

[7] Inoue M, Kyo S, Fujita M, Enomoto T, Kondoh G (1994). Coexpression of the c-kit receptor and the stem cell factor in gynecological tumors. Cancer Res.

[8] Krystal GW, Hines SJ, Organ CP (1996). Organ. Autocrine growth of small cell lung cancer mediated by coexpression of c-kit and stem cell factor. Cancer Res.

[9] Pietsch T, Kyas U, Steffens U, Yakisan E, Hadam MR, Ludwig WD, Zsebo K, Welte K (1992). Effects of human stem cell factor (c-kit ligand) on proliferation of myeloid leukemia cells: Heterogeneity in response and synergy with other hematopoietic growth factors. Blood.

[10] Toyota M, Hinoda Y, Takaoka A, Makiguchi Y, Takahashi T, Itoh F, Imai K, Yachi A (1993). Expression of c-kit and kit ligand in human colon carcinoma cells. Tumour Biol.

[11] Kindblom LG, Remotti HE, Aldenborg F, Meis-Kindblom JM (1998). Gastrointestinal pacemaker cell tumor (GIPACT): gastrointestinal stromal tumors show phenotypic characteristics of the interstitial cells of Cajal. Am J Pathol.

[12] Heinrich MC, Corless CL, Demetri GD, Blanke CD, von Mehren M, Joensuu H, McGreevey LS, Chen CJ, Van den Abbeele AD, Druker BJ, Kiese B (2003). Kinase mutations and imatinib response in patients with metastatic gastrointestinal stromal tumor. J Clin Oncol.

[13] Hirota S, Isozaki K, Moriyama Y, Hashimoto K, Nishida T, Ishiguro S, Kawano K, Hanada M, Kurata A, Takeda M, Muhammad Tunio G (1998). Gain-of-function mutations of c-kit in human gastrointestinal stromal tumors. Science.

[14] Rubin BP, Singer S, Tsao C, Duensing A, Lux ML, Ruiz R, Hibbard MK, Chen CJ, Xiao S, Tuveson DA, Demetri GD (2001). KIT activation is a ubiquitous feature of gastrointestinal stromal tumors. Cancer Res.

[15] Wardelmann E, Losen I, Hans V, Neidt I, Speidel N, Bierhoff E, Heinicke T, Pietsch T, Büttner R, Merkelbach-Bruse S (2003). Deletion of Trp-557 and Lys-558 in the juxtamembrane domain of the c-kit protooncogene is associated with metastatic behavior of gastrointestinal stromal tumors. Int J Cancer.

[16] Emile JF, Théou N, Tabone S, Cortez A, Terrier P, Chaumette MT, Julié C, Bertheau P, Lavergne-Slove A, Donadieu J, Barrier A (2004). A clinicopathologic, phenotypic, and genotypic characteristics of gastrointestinal mesenchymal tumors. Clin Gastroenterol Hepatol.

[17] Debiec-Rychter M, Sciot R, Le Cesne A, Schlemmer M, Hohenberger P, van Oosterom AT, Blay JY, Leyvraz S, Stul M, Casali PG, Zalcberg J, EORTC Soft Tissue and Bone Sarcoma Group; Italian Sarcoma Group; Australasian GastroIntestinal Trials Group (2006). KIT mutations and dose selection for imatinib in patients with advanced gastrointestinal stromal tumours. Eur J Cancer.

[18] Heinrich MC, Corless CL, Blanke CD, Demetri GD, Joensuu H, Roberts PJ, Eisenberg BL, von Mehren M, Fletcher CD, Sandau K, McDougall K (2006). Molecular correlates of imatinib resistance in gastrointestinal stromal tumors. J Clin Oncol.

[19] Demetri GD, von Mehren M, Blanke CD, Van den Abbeele AD, Eisenberg B, Roberts PJ, Heinrich MC, Tuveson DA, Singer S, Janicek M, Fletcher JA (2002). Efficacy and safety of imatinib mesylate in advanced gastrointestinal stromal tumors. N Engl J Med.

[20] Bono P, Krause A, von Mehren M, Heinrich MC, Blanke CD, Dimitrijevic S, Demetri GD, Joensuu H (2004). Serum KIT and KIT ligand levels in patients with gastrointestinal stromal tumors treated with Imatinib. Blood.

[21] Hirano K, Shishido-Hara Y, Kitazawa A, Kojima K, Sumiishi A, Umino M, Kikuchi F, Sakamoto A, Fujioka Y, Kamma H (2008). Expression of stem cell factor (SCF), a KIT ligand, in gastrointestinal stromal tumors (GISTs): a potential marker for tumor proliferation. Pathol Res Pract.

[22] Théou-Anton N, Tabone S, Brouty-Boyé D, Saffroy R, Ronnstrand L, Lemoine A, Emile JF (2006). Co expression of SCF and KIT in gastrointestinal stromal tumours (GISTs) suggests an autocrine/paracrine mechanism. Br J Cancer.

[23] Duensing A, Medeiros F, McConarty B, Joseph NE, Panigrahy D, Singer S, Fletcher CD, Demetri GD, Fletcher JA (2004). Mechanisms of oncogenic KIT signal transduction in primary gastrointestinal stromal tumors (GISTs). Oncogene.

[24] Lux ML, Rubin BP, Biase TL, Chen CJ, Maclure T, Demetri G, Xiao S, Singer S, Fletcher CD, Fletcher JA (2000). KIT extracellular and kinase domain mutations in gastrointestinal stromal tumors. Am J Pathol.

[25] Théou N, Tabone S, Saffroy R, Le Cesne A, Julié C, Cortez A, Lavergne-Slove A, Debuire B, Lemoine A, Emile JF (2004). High expression of both mutant and wild-type alleles of c-KIT in gastrointestinal stromal tumors. Biochim Biophys Acta.

[26] Pedersen M, Rönnstrand L, Sun J (2009). The c-Kit/D816V mutation eliminates the differences in signal transduction and biological responses between two isoforms of c-Kit. Cell Signal.

[27] Tabone-Eglinger S, Subra F, El Sayadi H, Alberti L, Tabone E, Michot JP, Théou-Anton N, Lemoine A, Blay JY, Emile JF (2008). KIT mutations induce intracellular retention and activation of an immature form of the KIT protein in gastrointestinal stromal tumors. Clin Cancer Res.

[28] Negri T, Bozzi F, Conca E, Brich S, Gronchi A, Bertulli R, Fumagalli E, Pierotti MA, Tamborini E, Pilotti S (2009). Oncogenic and ligand-dependent activation of KIT/PDGFRA in surgical samples of imatinib-treated gastrointestinal stromal tumours (GISTs). J Pathol.

[29] Hofmann UB, Kauczok-Vetter CS, Houben R, Becker JC (2009). Overexpression of the KIT/SCF in uveal melanoma does not translate into clinical efficacy of imatinib mesylate. Clin Cancer Res.

